# Self-Expandable Metal Stents in the Treatment of Acute Esophageal Variceal Bleeding

**DOI:** 10.1155/2011/910986

**Published:** 2011-10-15

**Authors:** Àngels Escorsell, Jaime Bosch

**Affiliations:** Intensive Care Unit and Hepatic Hemodynamic Lab, Liver Unit, Hospital Clínic IDIBAPS, University of Barcelona and Centro de Investigación Biomédica en Red de Enfermedades Hepáticas y Digestivas (CIBERehd), 08036 Barcelona, Spain

## Abstract

Acute variceal bleeding (AVB) is a life-threatening complication in patients with cirrhosis. Hemostatic therapy of AVB includes early administration of vasoactive drugs that should be combined with endoscopic therapy, preferably banding ligation. However, failure to control bleeding or early rebleed within 5 days still occurs in 15–20% of patients with AVB. In these cases, a second endoscopic therapy may be attempted (mild bleeding in a hemodynamically stable patient) or we can use a balloon tamponade as a bridge to definitive derivative treatment (i.e., a transjugular intrahepatic portosystemic shunt). Esophageal balloon tamponade provides initial control in up to 80% of AVB, but it carries a high risk of major complications, especially in cases of long duration of tamponade (>24 h) and when tubes are inserted by inexperienced staff. Preliminary reports suggest that self-expandable covered esophageal metallic stents effectively control refractory AVB (i.e., ongoing bleeding despite pharmacological and endoscopic therapy or massive bleeding precluding endoscopic therapy) with a low incidence of complications. Thus, covered self-expanding metal stents may represent an alternative to the Sengstaken-Blakemore balloon for the temporary control of bleeding in treatment failures. Further studies are required to determine the role of this new device in AVB.

## 1. Introduction

Acute variceal bleeding (AVB) is a severe complication of portal hypertension causing 70% of all upper gastrointestinal bleeding episodes in patients with portal hypertension [1]. Bleeding-related death, defined as any death occurring within six weeks of hospital admission for AVB, has decreased from 42% to 15–20% in the last two decades [2]. Prognostic factors for death include the severity of AVB (mainly failure to control bleeding and/or presence of early rebleeding), the degree of hepatic dysfunction (Child class C and/or MELD score ≥ 18 are associated with bad prognosis) and the development of complications, such as acute renal failure, bacterial infections, liver decompensation or of acute-on-chronic liver failure [2, 3]. 

## 2. Treatment of Acute Variceal Bleeding

Therapy of AVB should be aimed at correcting hypovolemia, preventing complications associated with gastrointestinal bleeding, and achieving hemostasis. Therefore, after initial general management including resuscitation, airway protection, and prevention of potential complications (including administration of prophylactic antibiotics), we must proceed to provide specific therapy and to confirm the diagnosis of the source of bleeding by emergency endoscopy, which should be performed within 12 hours from arrival to hospital. 

**Figure 1:**
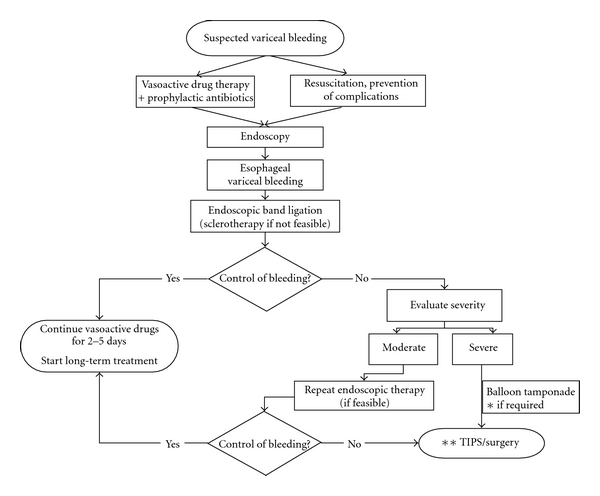
Algorithm of the current management of acute bleeding from ruptured esophageal varices. *Self-expandable esophageal metallic stent may represent an alternative to balloon tamponade in this situation. **Preferably TIPS with PTFE-covered stents (from reference [2], with permission).

Primary therapy of AVB includes early administration of vasoactive drugs (terlipressin, somatostatin, octreotide, or vapreotide) that should be combined with endoscopic therapy preferably esophageal banding ligation (EBL) once emergency endoscopy confirms bleeding from varices [3].  Figure 1 shows the algorithm of the management of AVB according to the Baveno V consensus conference. 

In 10 to 20% of patients, AVB is not controlled with this primary endoscopic and pharmacological therapy (failure to control bleeding or early rebleeding) [4]. 

To reduce the incidence of treatment failure and death, more aggressive therapies may be used in patients at high risk of treatment failure. In this sense, a recent randomized controlled trial has shown that early transjugular intrahepatic portosystemic shunt (TIPS) using PTFE-covered stents within 72 hours (preferably within ≤24 hours) markedly reduced treatment failure as well as short-term and 1-year mortality in high-risk patients (those Child class C < 14 points or Child with active bleeding despite being vasoactive drug therapy). Control patients received the standard therapy plus rescue TIPS in case of treatment failure. The Baveno V consensus conference recommended that “early >TIPS should be considered in patients at high risk of treatment failure (e.g., Child class C < 14 points or Child class B with active bleeding) after initial endoscopic and pharmacological therapy” [5].

## 3. Rescue Therapies in Acute Variceal Bleeding

Experts agree that TIPS with PTFE-coated stents is the treatment of choice in most patients who fail medical and endoscopic therapy [3]. However, “rescue” TIPS albeit very effective in controlling the bleeding is associated with a very high mortality (25–60%) due to the fact that the clinical condition of the patients deteriorates during the days in which bleeding is not controlled. Moreover, emergency TIPS could not always be performed, due to either lack of medical resources on a 24-hour basis or to the actual conditions of the patient precluding the immediate procedure. If possible (mild bleed in a hemodynamically stable patient), a second endoscopic therapy may be attempted. If this fails, or if bleeding is severe, balloon tamponade can be used. Consensus was reached that balloon tamponade should ONLY be used in massive bleeding as a temporary “bridge” until definitive treatment could be instituted (for a maximum of 24 hours, preferably under intensive care facilities) [3]. 

**Figure 2:**
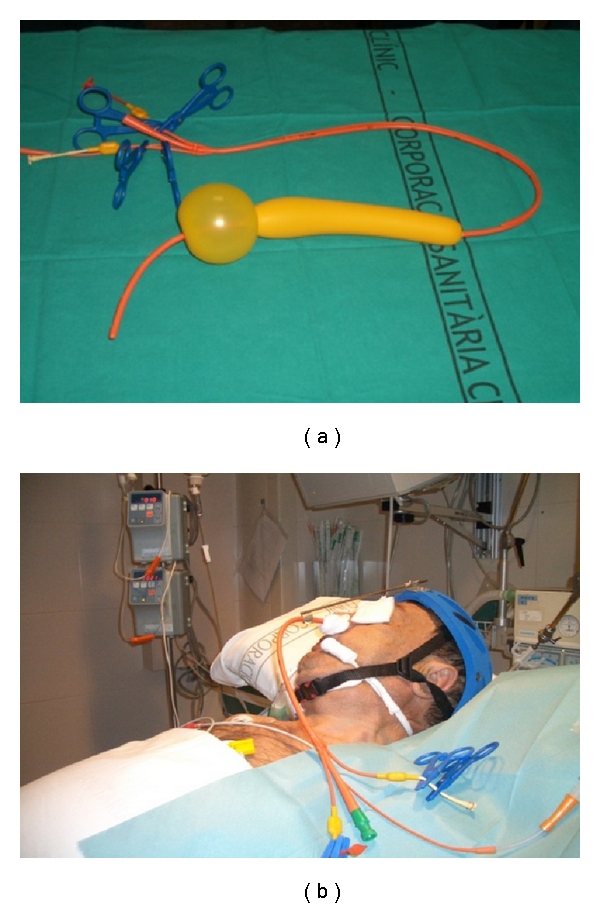
(a) Four lumen Sengstaken-Blakemore (Minnesota) tube. The gastric balloon is inflated with 150–200 mL of air and then pulled on the cardioesophageal junction and secured by different options such as a specially designed helmet (b). The esophageal balloon is inflated with air to a pressure of 40–50 mmHg. As shown, the tube has four lumen: the gastric and esophageal balloon inflation ports and the gastric and esophageal aspiration ports.

Balloon tamponade is aimed at obtaining hemostasis by direct compression of the bleeding varices. The type of the balloon varies according to the site of bleeding: the Linton-Nachlas tube in gastric fundal varices and the Sengstaken-Blakemore tube (Figure 2) in esophageal variceal bleeding. In experienced hands it provides initial bleeding control rates up to 80–90% [3, 6, 7], but recurrence is observed in about half of the patients after deflation of the balloon [7]. Major complications have been observed in over one fourth of the patients (fatal in 5% of the cases, especially in those of esophageal perforation), and its incidence increases with duration of tamponade [7] and when tubes are inserted by inexperienced staff [8]. Therefore, tamponade should only be used by skilled and experienced personnel [9] in intensive care facilities and with special caution in patients with respiratory failure or cardiac arrhythmias. Because of the high risk of aspiration pneumonia, tamponade should be preceded by prophylactic orotracheal intubation in comatose or encephalopathic patients.

In this context, preliminary studies have evaluated the use of self-expandable covered esophageal metallic stents as an alternative to balloon tamponade for the temporary control of bleeding in treatment failures.

## 4. Self-Expandable Metal Stents in the Treatment of Acute Esophageal Variceal Bleeding

**Figure 3:**
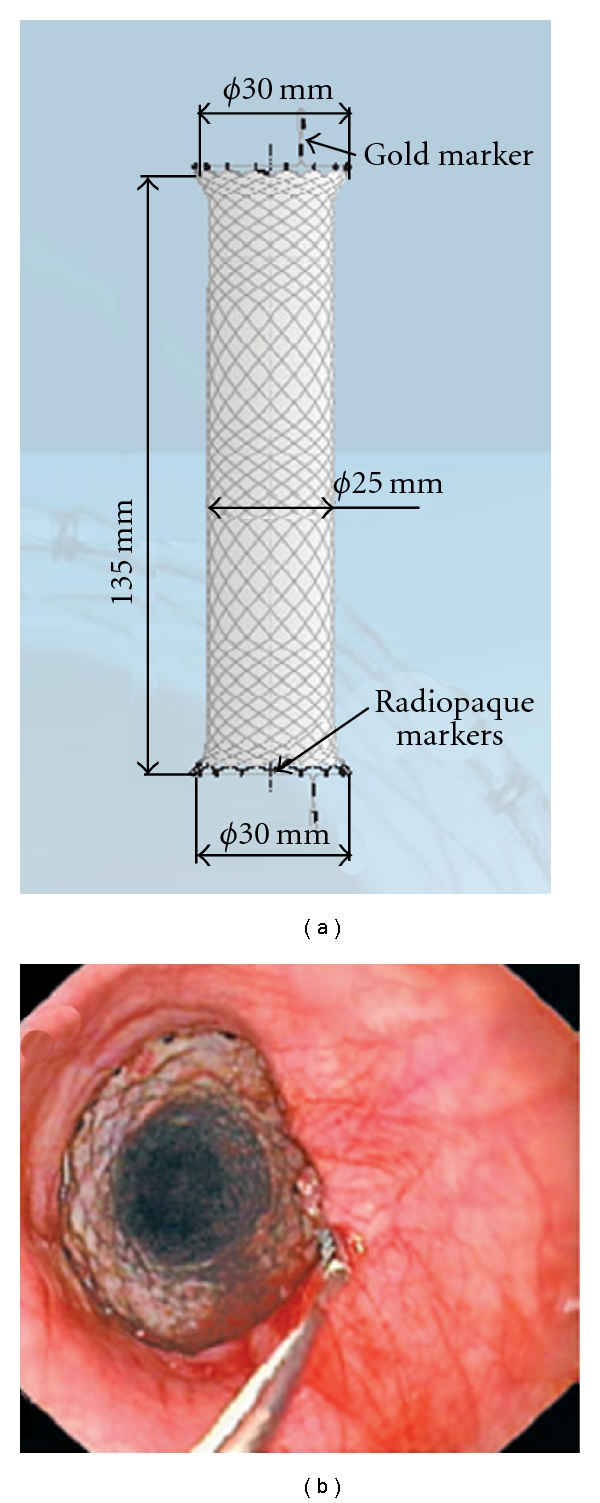
(a) Design of the SX-Ella Danis stent including radiopaque markers as well as the gold markers for stent removal. (b) Endoscopic view of the esophagus with the proximal end of the stent showing the extraction loop (gold marker).

The SX-Ella Danis stent (Ella-CS, Hradec Kralove, Czech Republic) is a removable, covered, and self-expanding metal stent that can be deployed in the lower esophagus without radiological or endoscopic assistance (Figure 3). The stent has atraumatic edges and radiopaque markers at both ends and at the midpoint to easily assess its position by a plain chest X-ray. Retrieval loops with gold markers at both stent ends allow the endoscopic extraction of the stent with a specifically designed system (PEX-Ella or Extractor for SX-Ella Stent Danis) (Figure 3).

Initial studies came from the center who developed the technique. Hubmann and coworkers reported the results in 20 patients with massive ongoing bleeding despite previous endoscopic treatment or balloon tamponade [10]. Interestingly, the first five patients received a nonspecifically designed esophageal stent (Choo stent and Ella-Boubela-Danis stent in 2 and 3 cases, respectively). Meanwhile, a new type of stent was specifically designed to treat bleeding esophageal varices (the SX-Ella Danis stent), and was used in the 15 remaining patients received. This pilot study showed that esophageal stenting for ABV was easily applicable (100% implantation success), sufficiently secure (except for 5 cases of migration of the stent to the stomach requiring further reposition), safe (no local complications were observed despite the stent remaining in place for 2 to 14 days) and highly effective, achieving hemostasis in all the patients included. The same authors presented recently an extended study, including 39 patients with active bleeding despite previous therapy [11]. The results were identical to those of their first report: stent insertion resulted in immediate hemostasis in 33 of 34 patients; further therapy (either endoscopic, radiological, or surgical) was possible in the majority of the patients; the only adverse event observed was stent migration in 7 patients, but this was not associated with bleeding, and the stent could be repositioned in all the cases by using the PEX-Ella extractor. 30-day survival was 74%. 

A group of British investigators published their experience in 10 patients with variceal hemorrhage with contraindications to TIPS insertion (due to extremely advanced liver failure, multiorgan failure, or hepatocellular carcinoma) or balloon tamponade (failure of balloon insertion or balloon-induced esophageal tear) [12]. The SX-Ella Danis stent insertion was successfully placed in 9 of the 10 patients, technically easy (without need for fluoroscopic control), effective (7 of 10 patients achieving hemostasis), safe (no major complications were observed) and could be used as an effective bridge to definite therapies (i.e., TIPS), since being in place for several days after achieving hemostasis allowed patient to improve enough as to be safely treated. Overall survival at 42 days in this short series of high-risk patients was 50%.

## 5. Self-Expandable Metal Stents versus Balloon Tamponade in the Treatment of Acute Esophageal Variceal Bleeding: Potential Indications, Limitations, Technical Requirements, and Complications

### 5.1. Indications

Potential indications for esophageal stenting not only relate to refractory variceal bleeding but also to cover large esophageal tears [12, 13], mainly induced by the Sengstaken-Blakemore tube, and banding or sclerotherapy-related ulcers [14].

Since the SX-Ella Danis stent could remain in place up to 7 days (and has been used for up to 14 days) [10–13], this may allow the patient to fully recover from the bleeding, unlike the Sengstaken-Blakemore balloon, that can be kept inflated for only 24 hours [3]. Therefore, the “extra” time in hemostasis given by the esophageal stent will permit some patients with relative contraindications to TIPS, such as hepatic encephalopathy, acute respiratory failure, and so forth, to improve enough to allow the safe placement of a TIPS or to go to other elective therapy without further bleeding [12].

If safety is confirmed in large series, it could also be proposed to use esophageal stents earlier in the course of bleeding, before declaring it “refractory” to standard therapy (for instance in high-risk patients needing to be transferred to another center for TIPS, or for patients with comorbidities precluding TIPS).

### 5.2. Limitations

Gastric varices will not be adequately compressed by the stent whereas the gastric balloon of the Sengstaken-Blakemore tube may have a role in some patients (GOV1 and some cases of GOV2). Nevertheless, it should be considered that most fundal varices (IGV1 and the majority of GOV2) will not be correctly treated by either the SX-Ella Danis stent or the Sengstaken-Blakemore tube. In these cases, endoscopic obturation with bucrylate is indicated, and tamponade with a Linton-Nachlas tube may be used in failures as a bridge to TIPS.

In patients with massive bleeding, in whom esophageal stent was deployed without previous diagnostic endoscopy, the persistence of bleeding strongly raises the suspicion of bleeding gastric varices [12]. In these cases, the insertion of a Linton-Nachlas tube, if therapeutic endoscopy is not feasible, should be considered.

### 5.3. Technical Requirements

Although both balloon tamponade and esophageal stenting could be deployed at the emergency room or the endoscopy room, the characteristics of the patients requiring such therapies advise to admit them to an intensive surveillance facility (intensive care unit, bleeding unit,…) attended by experienced physicians and nurses.

In this sense, it should be noted that therapy of ABV is not only directed at achieving hemostasis, but also at preventing bleeding-related complications (aspiration pneumonia, bacterial infections, renal failure, hepatic encephalopathy, etc.) [2, 3].

The technical requirements to insert either a Sengstaken-Blakemore tube or an SX-Ella Danis stent are scarce. In fact, both devices could be inserted and inflated/deployed without radiological or endoscopic assistance. It should be taken into account that most of the published cases of esophageal stents to treat AVB were deployed in the lower esophagus over an endoscopically placed guide wire [10–12] although, in our personal experience, this has not been necessary in patients without special esophageal conditions (hiatal hernia, strictures, lacerations …).

Figure 2 shows the step-by-step diagnostic and therapeutic algorithm in AVB. As shown, vasoactive therapy should be initiated as soon as possible followed by diagnostic endoscopy (in a hemodynamically stable patient). The initial endoscopy must confirm the source of bleeding, assess the severity of AVB and risk factors for failure to control bleeding, and evaluate the anatomy of the esophagus. During this procedure, endoscopic therapy, preferably banding ligation, must be performed. If the presence of active or massive bleeding precludes endoscopic therapy, we can choose between retiring the endoscope and inserting a Sengstaken-Blakemore tube or introducing a guide wire through the endoscope and afterwards use it to facilitate the insertion of the SX-Ella Danis stent. Unfortunately, in some cases, the bleeding is so as to preclude endoscopy, and we have to proceed to emergency tamponade without the possibility of confirming the source of bleeding, of discarding the presence of a giant hiatal hernia or of esophageal strictures, and without introducing a guide wire. 

After the placement of both the balloon or the stent, a chest radiograph must be taken to confirm that the device is correctly positioned.

### 5.4. Potential Complications

As previously mentioned, balloon tamponade can be associated with severe complications the most dreadful being esophageal perforation [7]. Usually, perforation occurs when the gastric balloon migrates or is initially inflated within the esophagus [15].

To avoid this often lethal complication, the SX-Ella Danis stent has a security pressure valve that does not allow the gastric balloon being inflated against resistance. This minimizes the risk of esophageal perforation in inexperienced hands. Moreover, as some authors described [12, 13], the covered esophageal stent achieved excellent results preventing mediastinitis and further bleeding in patients with esophageal perforations induced by a previous attempt at balloon tamponade. 

Fortunately, esophageal perforation is not the most frequent complication of balloon tamponade. This first position is reserved for aspiration pneumonia. The high incidence of this complication (47% in our unit) leads to the recommendation of protecting the patient's airway by means of endotracheal intubation whenever a Sengstaken-Blakemore tube is in place. Aspiration in many cases occurs during placement of the Sengstaken-Blakemore tube in an actively bleeding patients; in others, it is due to the fact that supraglottic secretions accumulate over the esophageal balloon and enter the tracheobronchial tree despite the presence of an esophageal aspiration port (Figure 2). Esophageal stents have the advantages of allowing the patient to swallow food (or an endoscope) without interfering with patient's airway. These advantages may result in a decreased risk of pulmonary aspiration and need for endotracheal intubation, and shortening the patient's ICU stage.

In addition, the presence of an esophageal stent is not a problem for maintaining oral nutrition, which is an essential component for the recovery of the patients.

## 6. Conclusions

According to reported data, the experts meeting at the Baveno V consensus conference agreed that “uncontrolled data suggest that self-expanding covered esophageal metal stent may be an option in refractory esophageal variceal bleeding” [3]. It should be clarified that “refractory esophageal variceal bleeding” refers to ongoing bleeding despite pharmacological and endoscopic therapy (except in massive bleeding precluding the latter). In these cases, the SX-Ella Danis stent would represent an alternative to the Sengstaken-Blakemore balloon.

Nevertheless, further studies, preferably randomized controlled trials, are required to determine the role of covered self-expanding metal stents in acute variceal bleeding.

## Abbreviations

AVB: Acute variceal bleeding
EBL: Esophageal banding ligation
GOV: Gastroesophageal varices
IGV: Isolated gastric varices
MELD: Model for end-stage liver disease
PTFE: Polytetrafluoroethylene
TIPS: Transjugular intrahepatic portosystemic shunt.

## References

[1] D’Amico G, de Franchis R (2003). Upper digestive bleeding in cirrhosis. Post-therapeutic outcome and prognostic indicators. *Hepatology*.

[2] Bosch J, Berzigotti A, Garcia-Pagan JC, Abraldes JG (2008). The management of portal hypertension: rational basis, available treatments and future options. *Journal of Hepatology*.

[3] Laine L, Abid S, Albillos A, de Franchis (2011). Treatment of acute bleeding. *Portal Hypertension V: Proceedings of the Fifth Baveno International Consensus Workshop*.

[4] Garcia-Tsao G, Sanyal AJ, Grace ND (2007). Prevention and management of gastroesophageal varices and variceal hemorrhage in cirrhosis. *Hepatology*.

[5] García-Pagán JC, Caca K, Bureau C (2010). Early use of TIPS in patients with cirrhosis and variceal bleeding. *New England Journal of Medicine*.

[6] D’Amico G, Pagliaro L, Bosch J (1995). The treatment of portal hypertension: a meta-analytic review. *Hepatology*.

[7] Avgerinos A, Armonis A (1994). Balloon tamponade technique and efficacy in variceal haemorrhage. *Scandinavian Journal of Gastroenterology*.

[8] Chojkier M, Conn HO (1980). Esophageal tamponade in the treatment of bleeding varices. A decadel progress report. *Digestive Diseases and Sciences*.

[9] D’Amico M, Berzigotti A, Garcia-Pagan JC (2010). Refractory acute variceal bleeding: what to do next?. *Clinics in Liver Disease*.

[10] Hubmann R, Bodlaj G, Czompo M (2006). The use of self-expanding metal stents to treat acute esophageal variceal bleeding. *Endoscopy*.

[11] Zehetner J, Shamiyeh A, Wayand W, Hubmann R (2008). Results of a new method to stop acute bleeding from esophageal varices: implantation of a self-expanding stent. *Surgical Endoscopy and Other Interventional Techniques*.

[12] Wright G, Lewis H, Hogan B, Burroughs A, Patch D, O’Beirne J (2010). A self-expanding metal stent for complicated variceal hemorrhage: experience at a single center. *Gastrointestinal Endoscopy*.

[13] Matull WR, Cross TJS, Yu D, Winslet MC, O’Beirne J (2008). A removable covered self-expanding metal stent for the management of Sengstaken-Blakemore tube-induced esophageal tear and variceal hemorrhage. *Gastrointestinal Endoscopy*.

[14] Mishin I, Ghidirim G, Dolghii A, Bunic G, Zastavnitsky G (2010). Implantation of self-expanding metal stent in the treatment of severe bleeding from esophageal ulcer after endoscopic band ligation. *Diseases of the Esophagus*.

[15] Hou MC, Lin HC, Chang FY, Lee FY, Chen TS, Lee SD (1994). Oesophageal perforation following endoscopic variceal ligation and balloon tamponade. *Journal of Gastroenterology and Hepatology*.

